# First- Versus Second- or Subsequent-Line Use of Cyclin-Dependent Kinase 4/6 Inhibitors for Hormone Receptor-Positive/Human Epidermal Growth Factor Receptor 2-Negative Metastatic or Recurrent Breast Cancer: A Multicenter, Retrospective Cohort Study

**DOI:** 10.3390/medicina62040661

**Published:** 2026-03-31

**Authors:** Shoko Yao, Shigeru Imoto, Ai Tsuchiya, Hirotsugu Isaka, Hirohito Seki, Sota Asaga, Shigehiro Yokoi, Kenji Koneri, Hiroyuki Maeda, Takanori Goi

**Affiliations:** 1 Department of Gastrointestinal and Breast Surgery, University of Fukui, 23-3 Matsuokashimoaizuki, Eiheiji-cho, Yoshida-gun, Fukui 910-1193, Japan; 2 Department of Breast Surgery, School of Medicine, Kyorin University, 6-20-2 Shinkawa, Mitaka-shi, Tokyo 181-8611, Japan; 3 Department of Surgery, Keiyu Hospital, 3-7-3 Minatomirai, Nishi-ku, Yokohama City 220-8521, Kanagawa, Japan

**Keywords:** breast cancer, hormone receptor-positive, HER2-negative, CDK4/6 inhibitors, palbociclib, abemaciclib, treatment sequencing

## Abstract

*Background and Objectives*: Cyclin-dependent kinase 4/6 inhibitors (CDK4/6i) combined with endocrine therapy are recommended as first- or second-line treatment for hormone receptor (HR)-positive, human epidermal growth factor receptor 2 (HER2)-negative advanced or recurrent breast cancer. However, in clinical practice, initiation timing varies according to patient characteristics, prior treatments, and the choice of the physician. Thus, the optimal timing of combination treatment remains unclear. *Materials and Methods*: In this multicenter retrospective cohort study, we reviewed 66 female patients who received CDK4/6i (palbociclib or abemaciclib) between March 2018 and November 2019. Patients were categorized into the first-line treatment (group A) (*n* = 21) and second- or subsequent-line treatment (group B) (*n* = 45) groups. In the latter group, endocrine therapy and/or chemotherapy had been administered previously. Duration of treatment with CDK4/6i (DOT), overall survival (OS), treatment duration of other regimens, and reasons for treatment discontinuation after CDK4/6i treatment were compared between groups. *Results*: The median DOT was significantly longer in group A than in group B (23 months (95% CI, 8–43) vs. 7 months (95% CI, 3–15); *p* = 0.015, at log-rank test). OS showed no significant difference between the two groups (*p* = 0.69, at log-rank test). *Conclusions*: In patients with HR-positive, HER2-negative advanced or recurrent breast cancer, first-line use of CDK4/6 inhibitors was associated with a significantly longer duration of treatment compared with second- or subsequent-line use. However, no significant difference in OS was observed between patients receiving CDK4/6 inhibitors as first-line or second- or subsequent-line therapy.

## 1. Introduction

Combination therapy with cyclin-dependent kinase 4 and 6 inhibitors (CDK4/6i) and endocrine therapy is recommended as first- or second-line treatment for hormone receptor (HR)-positive, human epidermal growth factor receptor 2 (HER2)-negative advanced or recurrent breast cancer, according to both the National Comprehensive Cancer Network Clinical Practice Guidelines in Oncology [1] and the American Society of Clinical Oncology Guideline Update [2].

Palbociclib was the first CDK4/6i to be approved for breast cancer treatment in the United States in February 2015 [3,4], followed by ribociclib in March 2017 [5] and abemaciclib in September 2017 [6,7].

CDK4/6i exert antitumor activity by targeting the restriction point (R point), a critical checkpoint in the G1–S phase transition of the cell cycle. In cancer cells, dysregulated growth factor or estrogen signaling promotes the formation of CDK4/6–cyclin D1 complexes, which phosphorylate the retinoblastoma (Rb) protein and release E2F transcription factors, thereby driving cell-cycle progression. By inhibiting CDK4/6 kinase activity, these agents prevent Rb phosphorylation, maintain R point control, and ultimately suppress tumor-cell proliferation [8,9,10].

Recently, clinical trials have examined treatment sequences in patients with HR-positive, HER2-negative advanced or recurrent breast cancer, comparing the use of first-line CDK4/6i to that of deferred therapy (Table 1) [11,12,13,14,15,16]. In these studies, compared with its implementation as second-line therapy, CDK4/6i demonstrated an absolute benefit in progression-free survival (PFS) when used as first-line, with most guidelines recommending the latter as the preferred strategy. Conversely, in the SONIA trial, PFS2 did not significantly differ between the two groups (median second progression-free survival, 31.0 vs. 26.8 months; hazard ratio [HR], 0.87; 95% confidence interval [CI], 0.74–1.03; *p* = 0.10), and no significant difference was observed in overall survival (OS) (median OS, 45.9 vs. 53.7 months; HR, 0.98; 95% CI, 0.80–1.20; *p* = 0.83). Thus, first-line use did not exhibit a survival benefit. Furthermore, grade ≥ 3 adverse events occurred more frequently with first-line administration, indicating the need for careful consideration before using CDK4/6i in this setting [11]. Nevertheless, most patients in the SONIA trial received palbociclib, making it difficult to evaluate the efficacy and safety profiles of other CDK4/6i, such as abemaciclib and ribociclib.

The SONIA trial demonstrated clinical validity for second-line combination treatment; nonetheless, the practical timing for using CDK4/6i often varies according to patient background and the choice of the physician.

In clinical practice, such treatment decisions may also be influenced by the site of recurrence, disease burden, patient characteristics such as age and socioeconomic circumstances, and concerns regarding tolerability and treatment cost. Therefore, it is important to evaluate the real-world treatment patterns of CDK4/6i and their association with clinical outcomes.

We conducted a multicenter, retrospective cohort study to compare prognostic outcomes of CDK4/6 inhibitor use as first- versus second- or subsequent-line therapy in patients with HR-positive/HER2-negative advanced or recurrent breast cancer (UMIN ID: UMIN000058459).

## 2. Materials and Methods

This study enrolled 66 patients with HR-positive/HER2-negative advanced or recurrent breast cancer who were treated with a CDK4/6i (abemaciclib or palbociclib) between March 2018 and November 2019 at the University of Fukui Hospital (*n* = 11) and Kyorin University Hospital (*n* = 55). The protocol was approved by the Institutional Review Board of University of Fukui Hospital (Approval No. 20250021) and Kyorin University Hospital (Approval No. 2723). This study was conducted in accordance with the Declaration of Helsinki.

Data were collected from the medical records, including clinicopathological and treatment-related information such as age, menopausal status, disease status (advanced or recurrent disease), metastatic sites, histological subtype, hormone receptor status, Ki-67 index, endocrine and chemotherapy treatment history, and treatment outcomes. In most patients, tumor markers, including carcinoembryonic antigen and cancer antigen 15-3, were examined every 1–3 months, and whole-body computed tomography scans were performed every 3–6 months. The tumor response was primarily assessed according to the Response Evaluation Criteria in Solid Tumors (RECIST) version 1.1 [17]. In some patients, the decision of physicians to continue or change treatment was based on tumor marker elevation or clinical findings.

Patients were categorized as follows: the first-line treatment group (group A) (*n* = 21), who received CDK4/6i as initial systemic therapy, and the second- or subsequent-line treatment group (group B) (*n* = 45), who had received prior endocrine therapy and/or chemotherapy before CDK4/6i administration.

OS was defined as the interval from the date of diagnosis of metastasis or recurrence to that of death from any cause. Duration of treatment (DOT) for CDK4/6i was defined as the time from initiation of therapy to documented disease progression, death, or treatment discontinuation for any reason.

### Statistical Analysis

Statistical analyses were performed using EZR (Easy R) version 1.68 (Saitama Medical Center, Jichi Medical University, Saitama, Japan), a graphical user interface for R [18]. Survival curves for DOT and OS were generated using the Kaplan–Meier method, and between-group comparisons were performed using the log-rank test. Categorical baseline characteristics of the patients and reasons for discontinuing post–CDK4/6i therapy were compared using Fisher’s exact test. Continuous variables, such as age, were compared using an unpaired *t*-test, whereas treatment durations and other non-normally distributed variables were compared using the Mann–Whitney U test. Statistical significance was set at a two-sided *p*-value < 0.05.

## 3. Results

### 3.1. Patient Characteristics

The patient characteristics are summarized in Table 2. The median patient age was 64 years, and all patients were women. The median follow-up period was 41 months (range, 1–81 months). CDK4/6i were administered as first-line therapy in 21 patients and second- or subsequent-line therapy in 45 patients (second-line therapy, *n* = 14; third-line therapy, *n* = 17; fourth-line therapy, *n* = 6; fifth-line therapy or beyond, *n* = 8).

Ten patients (15%) were premenopausal, while 56 (85%) were postmenopausal. Twenty-four (36%) and 42 (64%) patients exhibited advanced breast cancer and recurrent disease, respectively. Visceral metastases were present and absent in 50 (76%) and 16 (24%) patients, respectively. Histopathological examination revealed invasive ductal carcinoma and invasive lobular carcinoma in 61 and five patients, respectively. All tumors were estrogen receptor-positive, and 55 were progesterone receptor-positive. The Ki-67 index was ≥30% in 10 patients among patients with available data.

Endocrine therapy included selective estrogen receptor modulators, selective estrogen receptor downregulators, aromatase inhibitors, and luteinizing hormone–releasing hormone agonists in 27, 59, 61, and 10 patients, respectively. Chemotherapy included anthracyclines, taxanes, and S-1 (an oral fluoropyrimidine) in 17, 22, and 26 patients, respectively.

First-line use was chosen owing to visceral metastases or at the discretion of the physician. Baseline patient characteristics were generally comparable between the groups; with differences observed in treatment history and certain biological factors, including the Ki-67 index.

### 3.2. DOT of CDK4/6i

**Figure 1:**
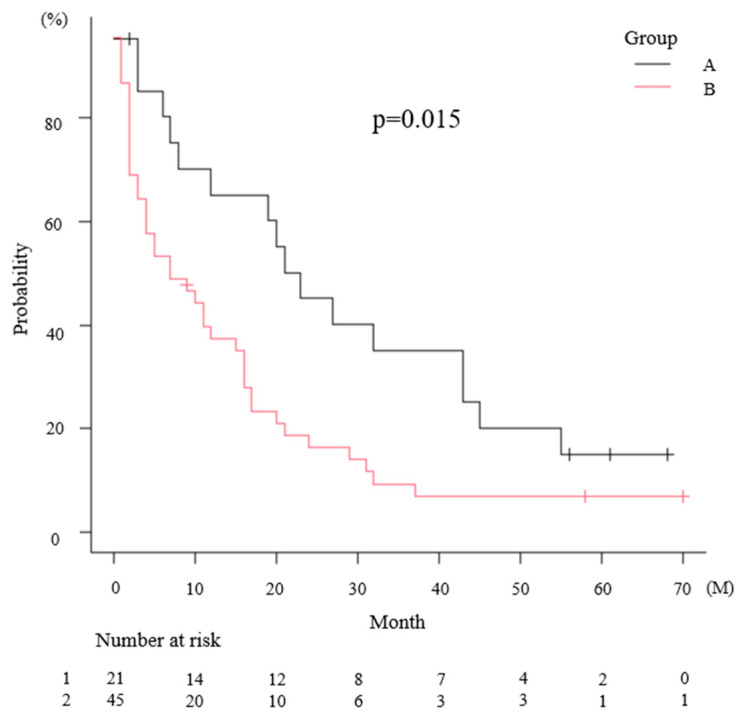
DOT of CDK4/6i Kaplan–Meier curves comparing Group A vs. Group B. The median DOT was 23 months (95% CI, 8–43) and 7 months (95% CI, 3–15) in groups A and B; *p* = 0.015. The shorter black and red lines indicate censored cases in Groups A and B, respectively.

Kaplan–Meier analysis showed a significant difference in DOT between the two groups (*p* = 0.015 at log-rank test). The median DOT was 23 months (95% CI, 8–43) and 7 months (95% CI, 3–15) in groups A and B, respectively (Figure 1).

### 3.3. Overall Survival

**Figure 2:**
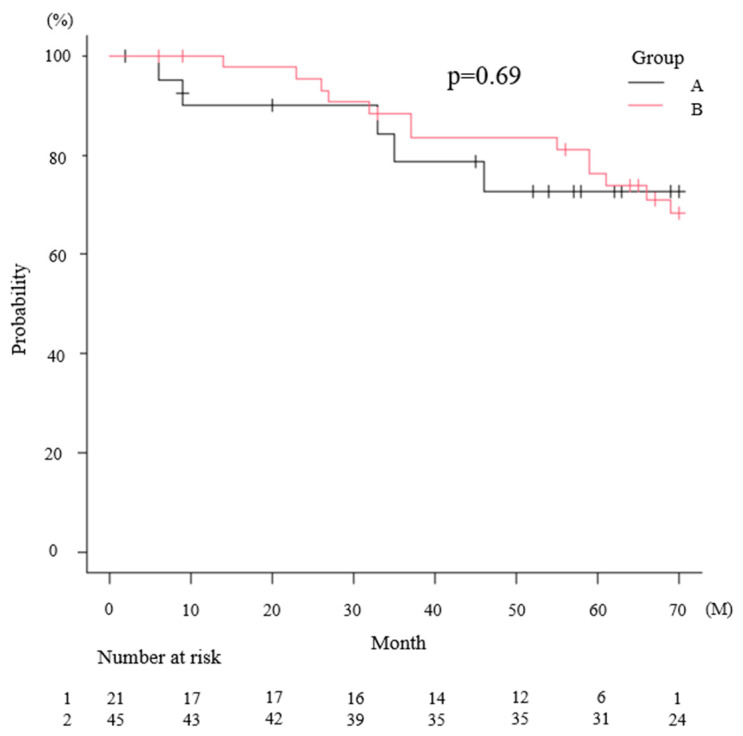
Overall Survival The 5-year OS rates were 72.7% in group A and 76.3% in group B. No significant difference in OS was observed between the two groups. The shorter black and red lines indicate censored cases in Groups A and B, respectively.

Kaplan–Meier analysis showed no significant difference in OS between the two groups (*p* = 0.69 at the log-rank test). The median OS was not reached in group A (95% CI, 46 months–not reached) and was 93 months (95% CI, 76–137) in group B. The 5-year OS rates were 72.7% (95% CI, 46.1–87.7) and 76.3% (95% CI, 60.4–86.5) in groups A and B, respectively (Figure 2).

### 3.4. Treatment Duration of Non–CDK4/6i Agents and Post–CDK4/6i Agents

**Figure 3:**
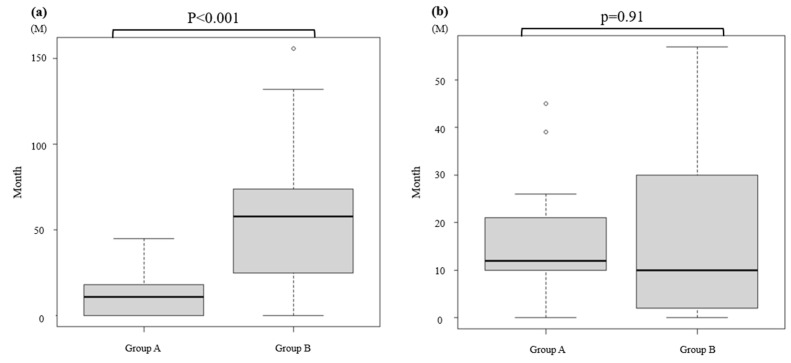
Treatment Duration of Non–CDK4/6i Agents (**a**) and Post–CDK4/6i Agents (**b**). (**a**) Median duration of non–CDK4/6i agents: 11 months (IQR, 0–18) and 58 months (IQR, 25–74) in Groups A and B; *p* < 0.001. (**b**) Median duration of post–CDK4/6i therapy: 12 months (IQR, 10–21) and 10 months (IQR, 2–30) in Groups A and B; *p* = 0.91. Circles indicate outliers, the horizontal lines within the boxes indicate medians, and the dotted lines extend to the non-outlier range.

For agents excluding CDK4/6i, the median treatment duration was 11 months (IQR, 0–18) and 58 months (IQR, 25–74) in groups A and B, respectively, with the latter being significantly longer than the former (*p* < 0.001) (Figure 3a).

The median duration of therapy administered after discontinuation of CDK4/6i was 12 months (IQR, 10–21) and 10 months (IQR, 2–30) in groups A and B, respectively, with no significant difference between the groups (*p* = 0.91) (Figure 3b).

Among the 36 patients who ultimately transitioned to best supportive care (BSC) (group A, *n* = 8; group B, *n* = 28), the primary reason for discontinuation of post-CDK4/6i therapy was progressive disease (PD) in seven (88%) and 22 (79%) patients in groups A and B, respectively (Table 3). Discontinuation due to adverse events (AEs) was recorded in one (13%) and six (21%) patients in groups A and B, respectively. Fisher’s exact test revealed no significant differences between the groups (*p* = 1.00). The outcome after transition to BSC was death in all patients.

AEs leading to discontinuation included myelosuppression (*n* = 3), liver dysfunction (*n* = 3), drug-induced interstitial pneumonia (*n* = 1), and anorexia (*n* = 2).

## 4. Discussion

Here, the duration of treatment with CDK4/6i was significantly longer in the first-line group than in the second- or subsequent-line group. However, despite this difference in DOT, OS did not differ significantly between the groups, regardless of the timing of CDK4/6i initiation. This finding may partly reflect the longer duration of non-CDK4/6i therapies administered in the second- or subsequent-line group, which may have contributed to the maintenance of OS.

Several studies have investigated whether CDK4/6i, in addition to an aromatase inhibitor, should be administered in the first-line setting (Table 1) [11,12,13,14,15,16]. While level I evidence supporting the clinical benefit of first-line CDK4/6i has been established, the control groups generally lacked a crossover design.

The SONIA trial, in which progression-free survival 2 (PFS2) was defined as the primary endpoint and OS as the secondary endpoint, found no significant differences in either PFS2 or OS between first- and second-line use of CDK4/6i. However, the duration of CDK4/6i treatment was substantially longer in the first-line group, with a median difference of 16.6 months compared with second-line use. Consistent with these findings, our study demonstrated that first-line administration of CDK4/6i was associated with a significantly longer duration of treatment compared with later-line use, with a median difference of approximately 16 months, while no significant difference in OS was observed between treatment lines.

In addition, first-line administration of CDK4/6i was associated with a higher incidence of grade ≥3 AEs: 83% of patients in the first-line group experienced at least one grade ≥3 event versus 64% in the second-line group, and the total numbers of grade ≥3 AEs were 2763 and 1591 in the first- and second-line groups, respectively, underscoring the greater cumulative toxicity burden of first-line CDK4/6i. Therefore, from a quality-of-life perspective, administering CDK4/6i as second-line therapy may be a reasonable option in selected patients. Moreover, using CDK4/6i as second-line therapy could reduce treatment duration, resulting in cost savings of €27,078 per patient, making this approach favorable from a health economics standpoint [11].

The most common reason for transition to BSC was PD, likely to reflect, in part, the development of resistance to CDK4/6i. In vitro and in vivo studies link such resistance to RB1 loss, CDKN2A (p16) amplification, mutations or amplification of fibroblast growth factor receptor 1 (FGFR1), and activation of the cyclin E–CDK2 complex. Because Rb is the direct target of CDK4/6i, its loss is a key driver of resistance. Overexpression of p16, an endogenous CDK4/6i, reduces available targets for CDK4/6i, promoting resistance. FGFR1 amplification activates the phosphatidylinositol 3-kinase/protein kinase B and rat sarcoma/mitogen-activated protein kinase kinase/extracellular signal-regulated kinase pathways. Cyclin E–CDK2 can phosphorylate Rb independently of CDK4/6, sustaining cell-cycle progression [19,20,21]. However, here, many patients had received prior therapies; thus, PD cannot be attributed solely to acquired resistance to CDK4/6i.

These findings may provide practical insight into treatment sequencing in patients with HR-positive/HER2-negative advanced or recurrent breast cancer. The timing for using CDK4/6i in routine clinical practice may depend on disease burden, prior treatment exposure, tolerability, and patient background. Additional real-world evidence is needed to further clarify the clinical positioning of CDK4/6i and to evaluate not only survival outcomes but also quality of life, toxicity burden, and treatment cost in routine practice.

The present study has some limitations. First, sampling bias may have arisen from physician preference. Second, lead-time bias may have occurred due to the irregular timing of examinations. Third, the criteria for dose reduction in cases of adverse events were not standardized. In addition, Ki-67 data were missing for some patients, which may have affected the interpretation of analyses involving this marker. Nevertheless, prior biomarker analyses of CDK4/6i therapy have not established Ki-67 as a robust predictor of treatment benefit; therefore, the influence of these missing data on the main findings may be limited [22]. Moreover, the study may have been underpowered to detect differences in OS. In addition, because OS was defined from the date of diagnosis of metastasis or recurrence, patients in the later-line group were required to survive until the initiation of CDK4/6i therapy, which may have introduced bias in the OS comparison. Accordingly, the absence of a statistically significant difference in OS should not be interpreted as evidence of non-inferiority, and the OS analysis should be considered exploratory. Some patients with a small tumor burden, such as those with locoregional recurrence, did not undergo treatment modification according to RECIST. To address this, we analyzed DOT rather than PFS. In a prior study, when a novel agent A was used as an experimental therapy or, alternatively, as a comparator to agent B, the duration of treatment with agent A tended to be shorter in the latter scenario [23]. Therefore, because a positive correlation between PFS and DOT was observed, DOT may serve as a surrogate indicator for estimating PFS.

## 5. Conclusions

First-line use of CDK4/6 inhibitors was associated with a significantly longer duration of treatment compared with second- or subsequent-line use. However, no significant difference in OS was observed between the two treatment groups.

## Figures and Tables

**Table 1:** First-line CDK4/6i trials and the SONIA sequencing trial in HR-positive/HER2-negative advanced or recurrent breast cancer.

Study	MONARCH3	PALOMA2	MONALEESA2	SONIA
Objective	1st-line treatment NSAI + ABE vs. NSAI (2:1)	1st-line treatment LET + PAL vs. LET (2:1)	1st-line treatment LET + RIB vs. LET (1:1)	1st-line CDK4/6i vs. 2nd- line CDK4/6i (1:1)
Patient background	Postmenopausal Visceral metastasis (53%)	Postmenopausal Visceral metastasis (49%)	Postmenopausal Visceral metastasis (59%)	Postmenopausal (86%) Visceral metastasis (56%)
Hormone receptor status	HR+ HER2-	HR+ HER2-	HR+ HER2-	HR+ HER2-
No. of patients	328 vs. 165	444 vs. 222	334 vs. 334	524 vs. 526
Primary endpoint	PFS: 28.2 mo vs. 14.8 mo HR 0.54; *p* < 0.001	PFS: 24.8 mo vs. 14.5 mo HR 0.58; *p* < 0.001	PFS: 25.3 mo vs. 16.0 mo HR 0.57; *p* < 0.001	PFS2: 31.0 mo vs. 26.8 mo HR 0.87; *p* = 0.10
Secondary endpoint	OS: 66.8 mo vs. 53.7 mo HR 0.75; *p* = 0.0664	OS: 53.9 mo vs. 51.2 mo HR 0.96; *p* = 0.34	OS: 63.9 mo vs. 51.4 mo HR 0.76; *p* = 0.008	OS: 45.9 mo vs. 53.7 mo HR 0.98; *p* = 0.83
Cross over	Not allowed	Not allowed	Not allowed	Protocol-defined crossover NSAI→CDK4/6i+FUL
Grade 3 or more AE	55% (180/327) vs. 22% (35/161)	76% (336/444) vs. 27% (54/222)	Not detailed (neutropenia 59.3% vs. 0.9%), elevated ALT (9.3% vs. 1.2%)	83% (433/520) vs. 64% (338/528)

ABE: abemaciclib, FUL: fulvestrant, NSAI: nonsteroidal aromatase inhibitor, LET: letrozole, PAL: palbociclib, RIB: ribociclib, ALT: alanine aminotransferase, PFS: progression-free survival, PFS2, second progression-free survival, OS: overall survival, HR+: hormone receptor–positive, HER2: human epidermal growth factor receptor 2 negative.

**Table 2:** Baseline demographics and clinical characteristics.

Factor		Group A	Group B	*p*-Value
Age, years (mean [SD])		63.81 (11.72)	63.20 (12.41)	0.85
Menopausal status (%)	Postmenopausal	17 (81.0)	39 (86.7)	0.71
	Premenopausal	4 (19.0)	6 (13.3)	
Histology (%)	IDC	18 (85.7)	43 (95.6)	0.32
	ILC	3 (14.3)	2 (4.4)	
Bone metastasis (%)	Absent	10 (47.6)	21 (46.7)	1.00
	Present	11 (52.4)	24 (53.3)	
Visceral metastasis (%)	Absent	4 (19.0)	12 (26.7)	0.56
	Present	17 (81.0)	33 (73.3)	
Liver metastasis (%)	Absent	12 (57.1)	25 (55.6)	1.00
	Present	9 (42.9)	20 (44.4)	
Lung metastasis (%)	Absent	14 (66.7)	30 (66.7)	1.00
	Present	7 (33.3)	15 (33.3)	
Disease status (%)	Recurrent	13 (61.9)	29 (64.4)	1.00
	Advanced	8 (38.1)	16 (35.6)	
Locoregional recurrence (%)	Absent	20 (95.2)	41 (91.1)	1.00
	Present	1 (4.8)	4 (8.9)	
ER status (%)	Positive	21 (100.0)	45 (100.0)	NA
PgR status (%)	Negative	2 (9.5)	9 (20.0)	0.48
	Positive	19 (90.5)	36 (80.0)	
HER2 status (%)	Negative	21 (100.0)	45 (100.0)	NA
Ki-67 index (%)	<30%	18 (94.7)	19 (67.9)	0.03
	≥30%	1 (5.3)	9 (32.1)	
AI use (%)	No	2 (9.5)	3 (6.7)	0.65
	Yes	19 (90.5)	42 (93.3)	
LHRH agonist use (%)	No	17 (81.0)	39 (86.7)	0.71
	Yes	4 (19.0)	6 (13.3)	
SERD use (%)	No	5 (23.8)	2 (4.4)	0.03
	Yes	16 (76.2)	43 (95.6)	
SERM use (%)	No	16 (76.2)	23 (51.1)	0.07
	Yes	5 (23.8)	22 (48.9)	
anthracycline (%)	No	20 (95.2)	29 (64.4)	0.01
	Yes	1 (4.8)	16 (35.6)	
taxane (%)	No	20 (95.2)	24 (53.3)	<0.01
	Yes	1 (4.8)	21 (46.7)	
S-1 (%)	No	16 (76.2)	24 (53.3)	0.11
	Yes	5 (23.8)	21 (46.7)	

IDC, invasive ductal carcinoma; ILC, invasive lobular carcinoma; ER, estrogen receptor; PgR, progesterone receptor; HER2, human epidermal growth factor receptor 2; AI, aromatase inhibitor; LHRH-agonist, luteinizing hormone–releasing hormone agonist; SERD, selective estrogen receptor downregulator; SERM, selective estrogen receptor modulator; NA, not available. *p* value for Ki-67 was calculated excluding patients with missing data (19 of 66 patients).

**Table 3:** Reasons for Discontinuation of Post–CDK4/6i Therapy.

Reason	Group A	Group B
PD (Progressive Disease)	7 (87.5)	22 (78.6)
AE (Adverse Events)	1 (12.5)	6 (21.4)
Total	8	28

## Data Availability

The data presented in this study are not publicly available due to privacy and ethical restrictions.
